# Transvenous lead extraction with laser reduces need for femoral approach during the procedure

**DOI:** 10.1371/journal.pone.0215589

**Published:** 2019-04-29

**Authors:** Arwa Younis, Michael Glikson, Amit Meitus, Noga Arwas, Sharon Shalom Natanzon, Dor Lotan, David Luria, Roy Beinart, Eyal Nof

**Affiliations:** 1 The Leviev Heart Center, Sheba Medical Center, Ramat Gan, Israel; 2 Sackler School of Medicine, Tel Aviv University, Tel Aviv, Israel; 3 Heart Center, Shaare Zedek Medical Center, Jerusalem, Israel; 4 Cardiovascular Research Institute Maastricht (CARIM), Maastricht University Medical Center, Limburg, the Netherlands; Indiana University, UNITED STATES

## Abstract

**Introduction:**

Cardiac implantable electronic device (CIED) trans venous lead extraction (TLE) is technically challenging. Whether the use of a laser sheath reduces complications and improves outcomes is still in debate. We therefore aimed at comparing our experience with and without laser in a large referral center.

**Methods:**

Information of all patients undergoing TLE was collected prospectively. We retrospectively compared procedural outcomes prior to the introduction of the laser sheath lead extraction technique to use of laser sheath.

**Results:**

During the years 2007–2017, there were 850 attempted lead removals in 407 pts. Of them, 339 (83%) were extracted due to infection, device upgrade/lead malfunction in 42 (10%) cases, and other (7%). Complete removal (radiological success) of all leads was achieved in (88%). Partial removal was achieved in another 6% of the patients. Comparison of cases prior to and after laser technique introduction, showed that with laser, a significantly smaller proportion of cases required conversion to femoral approach [31/275 (6%) laser vs. 40/132 (15%) non-laser; p<0.001]. However, success rates of removal [259/275 (94%) vs. 124/132 (94%) respectively; p = 0.83] and total complication rates [35 (13%) vs. 19 (14%) respectively; p = 0.86] did not differ prior to and after laser use. In multivariate analysis, laser-assisted extraction was an independent predictor for no need for femoral extraction (OR = 0.39; 95% CI 0.23–0.69; p = 0.01).

**Conclusion:**

Introduction of laser lead removal resulted in decreased need to convert to femoral approach, albeit without improving success rates or preventing major complications.

## Introduction

With the steady increase in the population life expectancy and the progress in medical knowledge and technology, the number of CIEDs (cardiovascular implantable electronic devices) implanted continues to rise worldwide [1, 2]. This results in an increase need for lead extraction above and beyond the rise in implantations [1, 3]. Leads are extracted due to infection, malfunction, venous stenosis, occlusion, need for device upgrade and more [4].

There are a large variety of tools available for an extractor. Options include locking stylets, mechanical and powered sheaths. In the last two decades the laser sheath has been introduced and is now widely used [5]. The decision which tool to use is made by the operator and the institute. Whether laser transvenous lead extraction (TLE) is preferable over non-laser (mechanical) methods, remains a challenging question. Several recent studies reported conflicting results [6–9]. However this has never been compared in a prospective randomized study. Our center is the largest referral center in our country. Since introduction of the laser sheath (January 2012) this has been the preferred method of TLE in our institute.

The goal of this study is to summarize our experience in the last decade, with the aim of specifically examine whether the use of laser sheaths improves efficacy and safety during extractions.

## Methods

### Study patients and design

**Fig 1:**
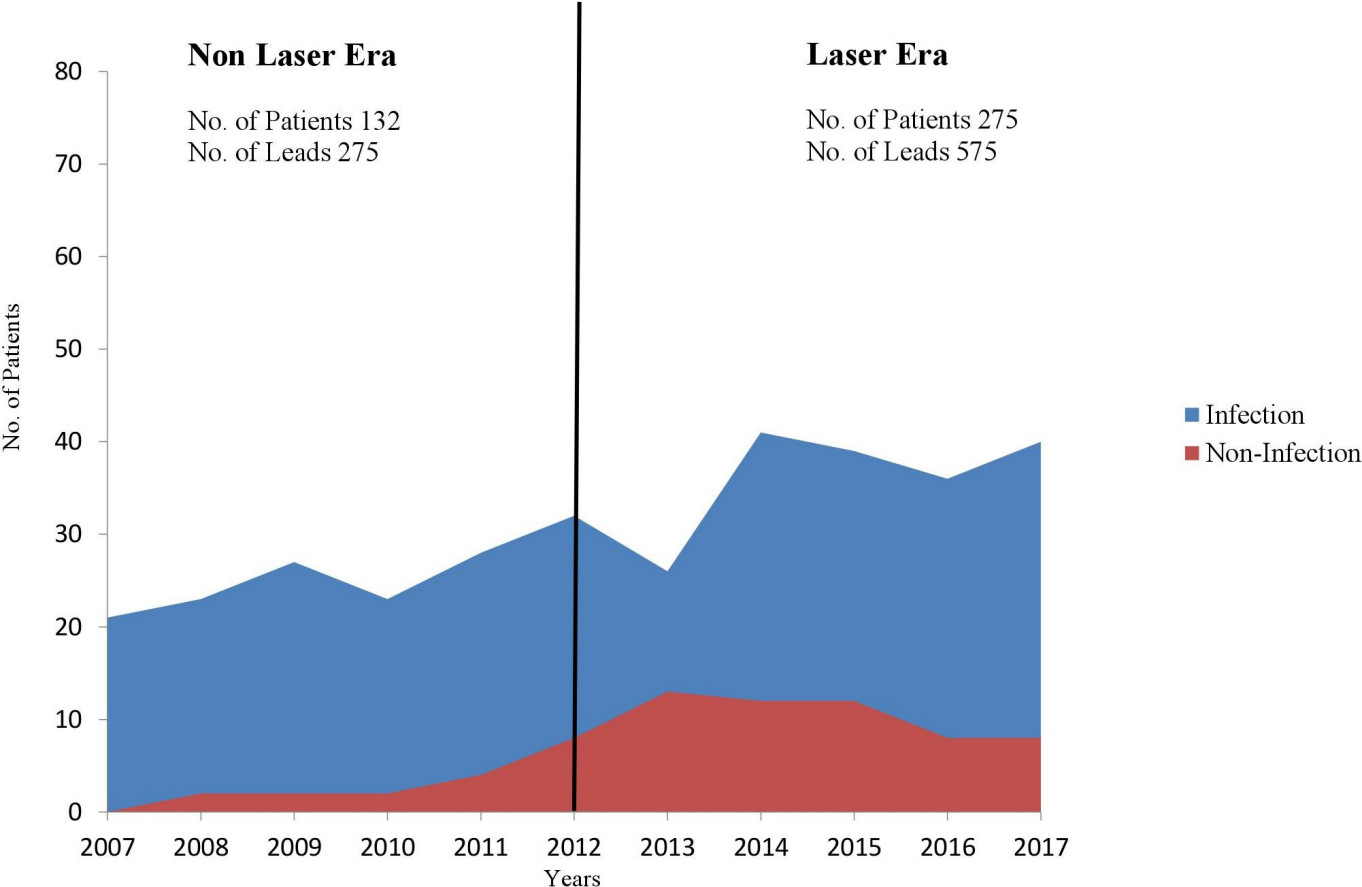
Trends in the case load and in the indications for lead extraction over the study period and times of introduction of new technology.

All consecutive patients who underwent TLE between January 2007 and October 2017 at our center were prospectively included. Group A (Non-Laser Era) comprised all consecutive patients until December 2011 (including), in whom mechanical sheath extraction was the first line strategy. Group B (Laser Era) comprised all consecutive patients who underwent TLE during the rest of the study period, in whom a change of our institute approach was performed (first line laser-assisted strategy). Please see Fig 1. All patients provided written informed consent. The study was approved by the Sheba International Review Board for human and animal trials Committee (IRB-Helsinki Committee).

### TLE procedure

All TLE procedures were performed with a cardiothoracic surgeon immediately available on site. Patients were under general anesthesia, with hemodynamic monitoring A transesophageal echocardiography probe was available in the room. A large-bore femoral venous access was inserted in all patients. The procedure was performed by qualified experienced operators (E.N, M.G, and D.L). A stepwise approach was used in all patients as following [10]:

1) Simple traction was applied on the lead from the pocket. For most cases, traction was performed after introduction of a locking stylet into the lead lumen (LLD, Spectranectics, Colorado Springs, Colorado) or Cook Liberator in the pre Laser era.
2A) During the Non-Laser Era; when removal by simple traction was not successful, we used at least 1 of the following mechanical tools: Evolution RL Controlled-Rotation Dilator Sheath, Teflon or Polypropylene Byrd Dilator (both are from Cook Medical, Bloomington, IN), stainless steel dilator, and electrosurgical dissection (EDS) sheath (Cook Vascular Incorporated, Vandergrift, PA, USA). The choice between these tools was left to the operator.
2B) During the Laser-Era; when removal by simple traction was not successful, we used the GlideLight Laser Sheath (Spectranetics, Colorado Springs, CO) as first option.
3) In both eras; in cases the lead was torn apart or could not be removed after a subclavian approach the femoral approach was attempted, using Needle’s Eye Snare Retrieval Set {Cook Medical, Leechburg, Pennsylvania}or Gooseneck snare {ev3, Europe SAS, Paris, France} or an ablation catheter.

The TLE procedure was terminated after complete removal of the leads, or when lead fragments could not be removed, or in the event of major complication.

### Procedural success and endpoints

Procedural outcome and success was defined in accordance with the 2017 HRS expert consensus [11];

- Complete Procedural Success: Removal of all targeted leads and all lead material from the vascular space, with the absence of any permanently disabling complication or procedure related death.
- Clinical Success: Removal of all targeted leads and lead material from the vascular space, or retention of a small portion of the lead (less than 4 cm) that does not negatively impact the outcome goals of the procedure. When the residual part does not increase the risk of perforation, embolic events, perpetuation of infection or cause any undesired outcome.
- Complications: were classified using the 2017 HRS conventional criteria and were attributed to the method used at the time the complication was observed. Complications were recorded until discharge from hospital.

### Statistical analysis

Continuous data are presented as mean ± standard deviation or as mean with range. Categorical data are presented as number (percentage). For univariate analysis, continuous variables were compared using the Mann-Whitney U test. Categorical variables were compared using the χ2 test or Fisher exact test, as appropriate.

Linear and logistic regression with robust standard errors was used to analyse the relations between the groups. Statistical significance was accepted for a 2-sided P value of <0.05. Statistical analysis was performed with SPSS version 21.0 (IBM Corp., Chicago, IL) and SAS version 9.2 (SAS Institute Inc., Cary, NC).

## Results

### Study population

During the last decade, a total of 407 patients underwent TLE. From January 2007 through December 2011, 132 patients underwent TLE (before laser era). Since the introduction of laser in our institute, January 2012, till December 2017, 275 patients underwent TLE (the laser era). Overall, 850 leads were extracted from the 407 patients. The mean age in both groups was similar (64 ± 16 years). Patients in the laser era group had significantly more comorbidities (Atrial fibrillation, hypertension, diabetes mellitus and current malignancy). Table 1.

**Table 1:** Baseline characteristics and indications for transcatheter lead extraction.

Variable	Before the Laser Era (No. 132)	Laser Era (275)	p-Value
Age (Years)	63 ± 17	65 ± 15	0.42
Male gender	103 (78%)	220 (80%)	<0.001
eGFR	65 ± 33	66 ± 33	0.37
Atrial Fibrillation	33 (25%)	95 (35%)	<0.001
Hypertension	63 (48%)	162 (59%)	<0.001
Past CHF Hospitalization	55 (41%)	127 (46%)	<0.001
Diabetes Mellitus	44 (33%)	117 (42%)	<0.001
Current Malignancy	3 (0.02%)	13 (0.05%)	<0.001
S.P Valve Replacement	14 (11%)	32 (12%)	0.08
**Indication for TLE**			
Pocket related	62 (48%)	86 (32%)	<0.001
Endocarditis/ Bacteremia	59 (45%)	132 (48%)	0.47
Lead malfunction/unused	4 (3%)	38 (13%)	<0.001
Other	7 (4%)	19 (7%)	<0.001
No. of leads	275	575	<0.001
No. of leads per patient	2.1	2.1	0.87
Lead dwelling time (years)	6 ± 3.8	8.2 ± 3.8	<0.001

CHF = congestive heart failure; eGFR = estimated glomerular filtration rate; S.P = status post; TLE = trans venous lead extraction.

The main indication for TLE in both groups was infection. Pocket related infection was significantly more common prior to laser era (48% vs. 32%, p<0.001, respectively). In contrast, TLE due to lead malfunction/avoiding abandoned leads was performed more during the laser era (13% vs 3%, p<0.001, respectively). The leads were significantly older during the laser era (mean lead dwelling time in years; 8.2 vs. 6, p<0.001, respectively). Detailed characteristics of the study groups are presented in Table 1.

### Procedural characteristics and success

The procedural characteristics and success rate of the two eras are detailed in Table 2. During the “laser era”, a higher proportion of leads (296 (51%)) were extracted using simple traction/ locking stylet than during the “before laser era” (93 (34%)) p<0.001 for the comparison. Cases of need to crossover from subclavian to femoral station were significantly more common prior to laser era (15% vs. 6%, p<0.001, respectively). The rates of both, radiological and clinical success were identical in both groups.

**Table 2:** Procedural techniques and outcomes.

Variable	Before the Laser Era (N = 275 leads)	Laser Era (N = 575 leads)	p-Value
Simple Traction	93 (34%)	296 (51%)	p<0.001
Laser	None	248 (43%)	None
Mechanical Sheath	142 (51%)	None	None
Femoral	40 (15%)	31 (6%)	p<0.001
Radiological Success	245 (89%)	500 (87%)	0.46
Clinical Success (per patient)	124 (94%)	259 (94%)	0.87

### Complications

The periprocedural complications during the two eras are detailed in Table 3. In both eras, there were similar rates of complications (14% during the “before laser era”, and 13% during the laser era, p = 0.86 for the comparison). There was a trend for periprocedural mortality (all due to superior vena cava (SVC) tear) during the “laser era” (3 (1.1%) patient’s vs. none, p = 0.24). As expected, SVC tear occurred only while using the laser sheaths {4 (1.5%) patients versus none with other sheaths (p = 0.13 for the comparison)}. During the “laser era”, 8 (3%) patients required pericardiocentesis due to significant pericardial effusion/tamponade, compared to 2 (1.5%) during the “before laser era”, p = 0.65.

**Table 3:** Complications during the two eras.

Variable	Before the Laser Era (N = 132 Patients)	Laser Era (N = 275 Patients)	p-Value
Total complications	19 (14%)	35(13%)	0.86
Periprocedural mortality (all due to SVC tear)	None	3 (1.1%)	0.24
SVC Tear without mortality	None	4 (1.5%)	0.13
Significant pericardial effusion*	2 (1.5%)	8 (3%)	0.65
Hematoma/ bleeding+	15 (11%)	19 (7%)	0.14
Tricuspid valve damage	2 (1.5%)	4 (1.5%)	0.91

* Resulting in need for Pericardiocentesis; + The loss of ≥ 2 grams Hgb.

### The use of the femoral approach

In 71 leads there was need to crossover to femoral station, all leads were extracted successfully (clinical success) using this approach Use of femoral approach was significantly higher in the pre laser era compared to laser era (15% vs. 6%, p<0.001, respectively).

Complications of the femoral approach were mainly minor bleedings and hematoma, there was no significant difference regarding complications rate among both eras.

Multivariate analysis (Table 4), based on the results of the univariate analysis/ clinical perspective including age, gender and infection, demonstrated that the “laser era” was associated with a decreased risk for the use of the femoral station with a HR of 0.39, 95% CI of 0.23–0.69, p = 0.01.

**Table 4:** Multivariate analysis predicting the need to crossover to the femoral approach.

Variable	HR	95% CI	p
Laser Era	0.39	0.23–0.69	0.01
Age ≥ 65 Years	0.68	0.33–1.41	0.32
Male Gender	0.82	0.43–1.60	0.57
Infection	0.67	0.27–1.65	0.38

## Discussion

The main finding of our study is that the use of laser significantly reduced the need to convert to femoral approach during the procedure without increasing the complication rates. However the overall procedural success did not improve with laser.

A comprehensive review of the literature from the recent decade makes it impossible to draw clear conclusions, but a certain trend is being repeated. Most studies demonstrate higher procedural success rates along with higher overall complication rates for laser extraction methods over non-laser methods, especially higher SVC tear rate [6–9].

A meta-analysis of 62 studies spanning a 15-year period and comprising 13,000 patients with 20,000 leads undergoing extraction, showed that the use of laser sheath was associated with an increased risk of major complications or death (p = 0.029) despite being associated with higher technical success of extraction (p = 0.003) [12].

An observational retrospective study including 1,449 patients who underwent laser-assisted lead extraction of 2,405 leads in 13 sites in the US and Canada between 2004–2007, showed higher procedural success rate and a similar procedural-related complication rate (with the exception of mortality rate, which was lower) [13].

A recent prospective study, which includes a large series of patients, summarizes the progress of mechanical lead-extraction methods over a decade. It concludes that newer mechanical methods may both improve success rate and lower complication rate. However, a greater need for a femoral approach may emerge while using these methods [14].

The above studies are not entirely in agreement with our findings in terms of success rates or complications. However our study did show that mechanical approach was an independent predictor for the need to convert to a femoral approach. Our study is the first to evaluate the rate of conversion to femoral approach thus significantly shortening the procedure. There was no significant evolution in mechanical tolls over the last decade thus differences between studies cannot be attributed to older tools.

The use of mechanical sheaths even powered ones obliges the operator to use more stress on the lead by pulling back the lead while advancing the sheath. The laser sheath allows relatively gentler traction as the main aim of pulling back it to keep a straight rail for the laser sheath.

This is the reason that less leads tear apart translating to lesser need for femoral approach. We do not have data on or measure the length of the procedure but there is no doubt that conversion to femoral approach will significantly lengthen the procedure. Thus in our experience since the use of laser TLE procedures shortened significantly.

One of the serious frequent major complications associated with laser-assisted lead extraction is SVC or innominate vein injury. Despite the awareness of this complication, and despite having cardiac surgeons present, this complication remains one with very high mortality rate reaching as high as 50% of the cases [13, 15]. However, vein tear is rarely seen while using the mechanical-assisted sheaths. Although our study failed to show significant differences in the rates of death or complications rate among the two different eras, yet we can clearly see a trend for more vascular injury and secondary death among the laser era. This observation failed to reach statistical significance mainly due to the small number of patients included in this cohort.

Both observations, the converting to the femoral station, and the vascular injury could be attributed to the power and efficacy of the laser sheaths.

## Limitations

The main limitation of our study is the non-randomized design. There are some significant differences in the characteristics of the patients and the indications for the procedure. These differences are e secondary to expanding the indications for the extraction (i.e. older patients with more comorbidities). However in a multivariable analysis the use of laser was still the only predictive variable. This can be found in Table 4. The design of the study cannot eliminate the fact that the ability to crossover to another sheath may have biased the results. However, since we used the same stepwise approach in all patients during each period, we assume that this bias was low. Our relative low number of patients with complication resulted in difficulties concluding statistical significance. We assume that for evaluating the differences in complication rates, a larger sample size is needed.

Lastly, our data are might be prone to practice effect; physicians are usually more experienced and specialized in the laser era. However we would like to state that the extractors (MG and DL) participating in the pre laser era gained a lot of experience prior to initiation of our study registry while the other extractor (EN) joined the group only after introduction of laser. Therefore we believe that experience did not have a bias effect on our conclusions.

## Conclusion

In our high volume center, introduction of laser lead removal resulted in lesser need to convert to femoral approach, albeit without improving success rates or preventing major complications.

**Ethic approval**—Ethics approval and consent to participate: The study was approved by the Sheba institute’s internal review board and was performed according to the principles expressed in the Declaration of Helsinki and the ethics policy of the Sheba Medical Center.

## Supporting information

S1 Protocol General study protocol. (DOCX)

S2 Protocol Current study protocl. (DOCX)
