# Supplementary material for: Transvenous lead extraction with laser reduces need for femoral approach during the procedure
Source: PLoS One. 2019 Apr 29;14(4):e0215589. doi: 10.1371/journal.pone.0215589 (PMC6488060; doi:10.1371/journal.pone.0215589)
Supplement: S1 Protocol — (DOCX) [file pone.0215589.s001.docx]

Study Protocol

22.12.2006

EXTRACTION-Tel Hashomer Registry

| Investigator | Arwa Younis, Michael Glikson, Roy Beinart, Eyal Nof |
| --- | --- |
| E-mail | [Arwa.Younis@sheba.health.gov.il](mailto:Arwa.Younis@sheba.health.gov.il) |
| Phone | 0544731936 |

With the growing recognition of the clinical need and wider indications for cardiovascular implantable electronic devices (CIED), the number of implant procedures has increased considerably. Consequently, the rate of complications related to these devices has also increased. Transvenous lead extraction (TLE) is the gold standard in the treatment of CIED-related infective complications and is often required in the management of lead malfunction. In Israel we are currently the only center that performs TLE. The EXTRACTION-Tel Hashomer Registry is the first large prospective registry of consecutive patients undergoing TLE in the middle east and managed by the EP research group from the Sheba Medical Center.

The primary objective of the registry is to evaluate the acute and long-term safety of TLE; the secondary objective is to describe the characteristics of the patients, the leads, the indications for TLE, and the tools and techniques currently used for TLE. Each patient will be followed up for 1 year consecutively from January 2007 to December 2017. The target is to achieve a sample size of at least 500 patients for statistical analysis. Data will be collected using a Web-based system. The independence of the registry, the consecutiveness of the patient enrolment, and the monitoring of the study are characteristics of this registry that will contribute to the scientific validity of the objectives to be achieved.
